# Urine testing for diabetic analysis

**Published:** 2015

**Authors:** Janet Marsden, Dianne Pickering

**Affiliations:** Nurse Advisor: *Community Eye Health Journal*, London, UK. Email: **J.Marsden@mmu.ac.uk**; Nurse Advisor (retired): *Community Eye Health Journal***dianne_logan@hotmail.com**

**Figure:**
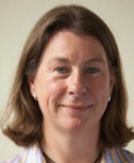
Janet Marsden

**Figure:**
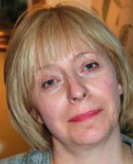
Dianne Pickering

Urine testing is relatively cheap and easy to do. Urine testing can be used to check for blood in the urine, to check for infection (by detecting the presence of white blood cells or protein) and can show up other systemic problems such as liver problems (by showing abnormal bilirubin levels). Urine testing can also detect ketones in the urine. Ketones are by-products of metabolism which form in the presence of severe high blood glucose. The presence of ketones in the urine therefore indicates that patients' blood glucose level is likely to be very high and that they may have ketoacidosis, which is a potentially life-threatening complication of diabetes and needs urgent treatment. Early signs of ketoacidosis include passing large amounts of urine, severe thirst, feeling nauseous, tiredness, abdominal pain and shortness of breath. Advanced signs include rapid breathing, rapid heartbeat, vomiting, dizziness, confusion and drowsiness; patients may even lose consciousness. Urgently refer patients with any of the above signs.

Although not as accurate as a blood glucose test, urine testing can be used as a screening tool in patients known to have diabetes. Even in patients with no ketoacidosis, high glucose levels may be an indication that their diabetes is poorly controlled. These patients can be referred for counselling, patient education, and-as soon as possible -for an eye examination to look for signs of diabetic retinopathy. Urine testing can also be used to detect glucose in the urine in undiagnosed patients; they will need to be referred for further tests and perhaps a diagnosis of diabetes. All patients with diabetes should have an eye examination once a year.

## Before you start

- Confirm that there is an standing order or request for the test to be conducted.
- Explain to the patient what you are going to do and why.

## What you need

- Personal protective equipment: gloves, eyewear (plus apron if available)
- Reagent strips – check the expiration date prior to use
- Reagent strip container with colour chart
- Clean container for collection of urine
- Optional: bedpan or bottle for patient unable to access a bathroom

**Note:** Reagent strips should be stored according to the manufacturer's instructions.

**Figure 1.**
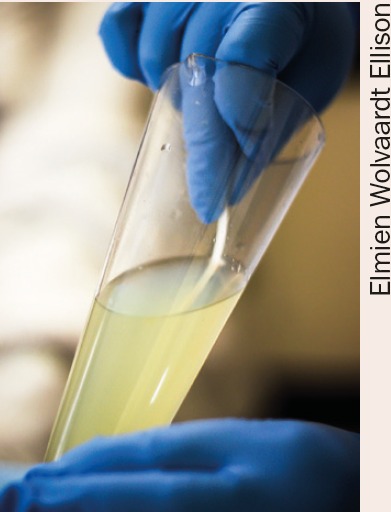
Completely immerse the reagent strip

**Figure 2.**
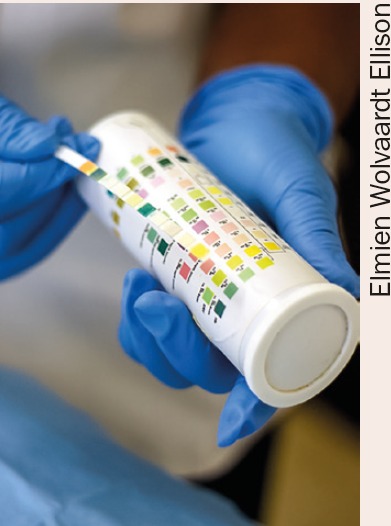
Read the strip against the colour chart on the container. Be careful not to allow the strip to touch the container

## Procedure

- Give the patient the clean container and explain to them how to obtain a clean specimen of urine. Remind them to wash their hands both before and after using the toilet.
- Depending on the patient, they can be asked to wipe around their genital area with a wet-wipe prior to sampling in order to ensure there are no external contaminants.
- If possible, ask the patient to urinate a little first before then urinating into the container. A mid-stream specimen most accurately represents the urine in the bladder.
- Let them know how much you need, i.e. fill the container three-quarters full, then place the lid on the top.
- If the patient is unable to perform this themselves, they will need assistance.
- Wash your hands and put gloves on prior to taking the container from the patient.
- Remove the lid and dip the reagent strip into the urine, completely immersing the strip in the urine (Figure 1). Remove immediately and tap on the side of the urine container to shake off the last drops.
- Hold the strip at an angle to allow any remaining urine to drain away.
- Wait the required time (as outlined on the reagent strip container) before determining the results by comparison with the colour chart on the side of the reagent strip container (Figure 2). Be careful not to touch anything, whether the side of the reagent strip container or any other surface.
- Dispose of the urine in an appropriate manner.
- Dispose of the contaminated equipment (gloves, reagent strip and urine container, if disposable) as your policy for clinical waste dictates.
- Remove gloves and wash hands.
- Record your readings in the patient's care notes.
- If readings are abnormal for the patient, pass the information on to someone who is responsible for the patient's care.

